# Structural and Optical Properties of Lead-Boro-Tellurrite Glasses Induced by Gamma-Ray

**DOI:** 10.3390/ijms14023201

**Published:** 2013-02-04

**Authors:** Iskandar Shahrim Mustafa, Halimah Mohamed Kamari, Wan Mohd Daud Wan Yusoff, Sidek Abdul Aziz, Azhar Abdul Rahman

**Affiliations:** 1 School of Physics, Universiti Sains Malaysia, 11800 Minden, Pulau Pinang, Malaysia; E-Mails: iskandarshah@usm.my (I.S.M.); arazhar@usm.my (A.A.R.); 2 Physics Department, Faculty of Science, Universiti Putra Malaysia, 43400 UPM, Serdang, Selangor, Malaysia; E-Mails: wmdaud@science.upm.edu.my (W.M.D.W.Y.); sidek@science.upm.edu.my (S.A.A.)

**Keywords:** tellurite glass, optical band gap, Urbach’s energy, irradiation

## Abstract

Spectrophotometric studies of lead borotellurite glasses were carried out before and after gamma irradiation exposure. The increasing peak on the TeO_4_ bi-pyramidal arrangement and TeO_3+1_ (or distorted TeO_4_) is due to augmentation of irradiation dose which is attributed to an increase in degree of disorder of the amorphous phase. The structures of lead tellurate contain Pb_3_TeO_6_ consisting of TeO_3_ trigonal pyramid connected by PbO_4_ tetragonal forming a three-dimensional network. The decrease of glass rigidity is due to irradiation process which is supported by the XRD diffractograms results. The decreasing values of absorption edge indicate that red shift effect occur after irradiation processes. A shift in the optical absorption edge attributed to an increase of the conjugation length. The values of optical band gap, *E*_opt_ were calculated and found to be dependent on the glass composition and radiation exposure. Generally, an increase and decrease in Urbach’s energy can be considered as being due to an increase in defects within glass network.

## 1. Introduction

Glass in an amorphous (non-crystalline) solid material. Glasses are typically brittle and optically transparent. The most familiar type of glass, used for centuries in windows and drinking vessels, is soda-lime glass, composed of about 75% silica (SiO_2_) plus sodium oxide (Na_2_O) from soda ash, CaO, and several minor additives. Some glasses that do not include silica as a major constituent may have physico-chemical properties useful for their application in fibre optics and other specialized technical applications. These include fluoride glasses, tellurite glasses, aluminosilicates, phosphate glasses, borate glasses and chalcogenide glasses. Tellurite glasses contain tellurium oxide (TeO_2_) as the main component. Tellurium dioxide is known as a conditional glass former, which it is, needs a modifier in order to easily form the glassy state. The formation of glass on two glass formers interest both scientific and practical locale. The structural network will be perturbed and may lead to the formation of new structural units [1,2]. Glass forming substances are fall into two categories of inorganic compounds containing bonds which are partially ionic and partially covalent, and, inorganic or organic compounds which form chain structures with covalent bonds within the chains and van der Waals’ bonds between the chains. Glasses containing heavy metal oxide (HMO) have recently attracted the attention of several researchers for the excellent infrared transmission compared with conventional glasses. The γ-irradiation on glasses is found to affect the optical and physical properties [3,4]. Hence, radiation damage caused by electrons, alpha particles and gamma rays has been thoroughly investigated [5]. The structural and physical properties of PbO glasses are well described by Worrel and Henshell [6]. In previous work, Atul *et al.* [7] have studied borate glasses containing heavy-metal oxides and shown that it has potential applications in radiation shielding. The objective of the present work is to study the effect of radiation on the structural and optical properties of lead borotellurite glass system. To achieve this, a systematic study on optical properties has been performed to understand the variation of irradiation dose as a function of PbO composition in borotellurite glasses. In addition, X-ray diffraction patterns and Raman spectra measurements were also performed in order to support the available data.

## 2. Results and Discussion

### 2.1. Raman Spectra

**Figure 1:**
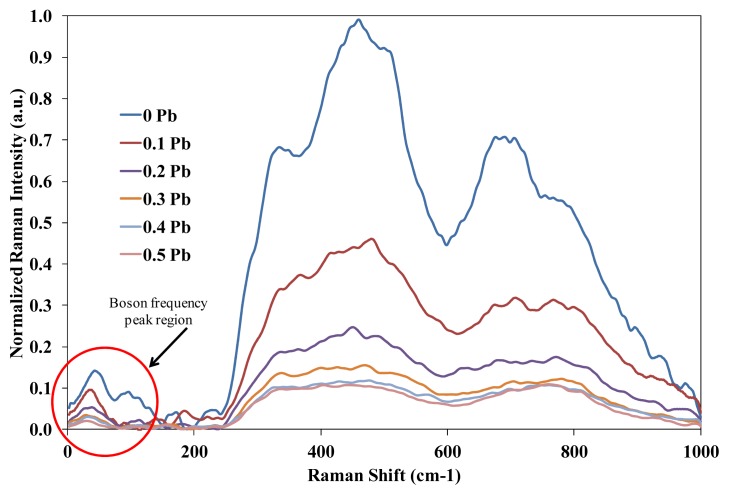
Raman spectra (at ambient temperature) of (TeO_2_)*_y_*[(PbO)*_x_*(B_2_O_3_)_1−_*_x_*]_1−_*_y_* glasses with different compositions before irradiation.

**Figure 2:**
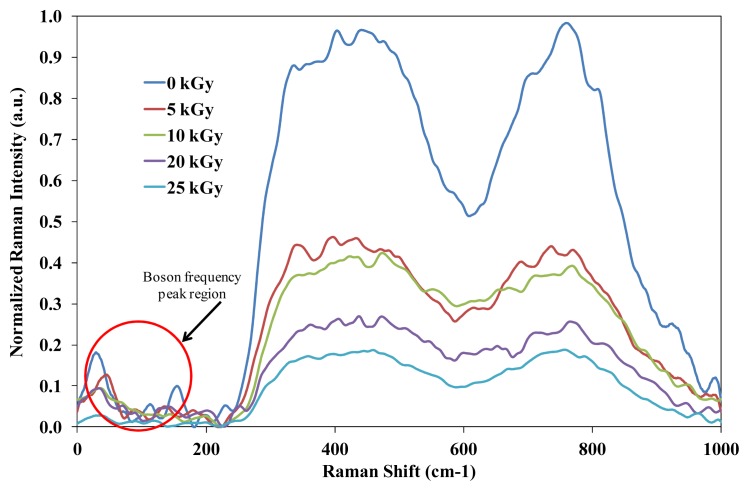
Raman spectra (at ambient temperature) of (TeO_2_)*_y_*[(PbO)*_x_*(B_2_O_3_)_1−_*_x_*]_1−_*_y_* glasses with different irradiation doses at *x* = 0.5% mol, *y* = 0.7% mol.

Marker labeling of Raman peak is shown in Table 1. Raman spectrum in Figure 1 and Figure 2 were corrected for baseline and normalized which allows for an effective comparison across a heterogeneous set of samples. Eventually, the baseline correction utilized the multiple point level method (Savitzgy-Golay) in which the baseline is leveled at a value that is the average of the baseline points. Normalization of Raman spectral was performed based on the common normalization method referring to min/max technique. The min/max (normalization) method is expressed by:

(1) $$I_{normal}=\frac{I-I_{min}}{I_{max}}$$

where *I* is the intensity after baseline correction was performed, *I_min_* is the minimum intensity and *I_max_* is the maximum intensity on single spectral measured. Raman spectrum in Figure 1 shows significant peak at <100 cm^−1^ which indicate the strong presence of Pb and Te in the chemical bonding through vibrational mode due to addition of PbO and glass network. PbO stands out as unique because of its dual role [8], one as modifier, if Pb–O bond is ionic and the other as glass former with PbO_4_ structural units, if Pb–O bond is covalent. Occasionally, PbO concentration deteriorates glass forming ability [9] of (TeO_2_)*_y_*[(PbO)*_x_*(B_2_O_3_)_1−_*_x_*]_1−_*_y_* system. The addition of heavy metal oxide modifiers to pure TeO_2_ leads to the progressive formation of distorted TeO_3+1_ polyhedron followed by the creation of regular trigonal TeO_3_ pyramids that contain non-bridging oxygen. In all compositions, the appearance of the low-frequency Boson peak (<200 cm^−1^) affirms the presence of the glass structure. The increase broad shoulders at 410 cm^−1^ indicate that new features to vibrations of one of the partially crystalline phase of Pb_3_TeO_6_. The existence of Pb_3_TeO_6_ is confirmed by X-ray analysis. Clearly, the shoulders at 410 cm^−1^ were getting broader as the content of PbO increased, possibly due to the PbO unique ability. At low portions of PbO (up to 0.2% mol), it enters the glass network by breaking up the Te–O–Te and B–O–B bonds and introduces coordinate defects known as dangling bonds along with non-bridging oxygen ions (Te–O^−^…Pb^2+^…^−^O–Te) which in turn neutralizes the negative charge of non-bridging oxygens (NBO) by forming TeO_3_ and BO_4_ units. Normally, the oxygen of PbO breaks the local symmetry while Pb^2+^ ions occupy interstitial positions. As PbO increases (from 0.3% to 0.5% mol), a considerable portion may be acts as a double bridges between adjacent TeO_4_ such as =Te–O–Pb–O–Te= which can formed besides the formation of PbO_4_ and TeO_3_ units. Therefore, for PbO ≥ 0.3% mol, Pb^2+^ acts as glass forming agent and is incorporated in the glass network in the form of PbO_4_ units. Decreasing on (ss) Te–O–Te and B–O–B bending shoulder at approximately 490 cm^−1^ and 450 cm^−1^ ascribe that the splitting of Te–O–Te and B–O–B bonds and hence, the bridging oxygen’s (BOs) are converted into NBOs. The pure B_2_O_3_ was known to consist of the boroxol rings by linking among trigonal-plane BO_3_ units, but the network structure was altered through the addition of PbO. Some parts of the boroxol ring of BO_3_ units were changed into BO_4_ tetrahedral units [10].

TeO_4_ trigonal bipyramids is known to be the main structural unit of the network of all tellurite glasses [11], as well as of the lattices of crystalline TeO_2_ polymorphs. All stable tellurite glasses are multi-component and, what is important, cations on non-tellurite components have a coordination number other than four. The tellurite structural units of two types are always present in multi-component tellurite glass network, namely, fourfold coordinated Te atoms (TeO_4_ trigonal bipyramids, where all O atoms from bridging bonds with the environment) and threefold coordinated Te atoms (O=TeO_2_ trigonal pyramids, where one of O atoms is non-bridging, one forms O=Te double bond and one O atoms form bridging bonds with the environment). The spectral features from 710 cm^−1^ to 730 cm^−1^ and 790 cm^−1^ in Figure 1 correspond to the TeO_4_ bi-pyramidal arrangement and the TeO_3+1_ (or distorted TeO_4_) and TeO_3_ trigonal pyramids structures respectively. It can be clearly observed that the evolution of TeO_4_ to TeO_3+1_ and TeO_3_ units which one of the Te sp3 hybrid orbital is occupied by a lone pair of electron. This transformation causes increases in the number of non-bridging oxygen (NBO) atoms. Legitimately, the increasing peak on the TeO_3_ trigonal pyramids shows that modification of lattice and interstitial occurs in the system due to addition of PbO and B_2_O_3_.

The presence peaks at <100 cm^−1^ in Figure 2 do not indicate any strong changes of intensity due to variation of gamma irradiation exposure. Eventually, the decrease in broad shoulders at 410 cm^−1^ also indicates the existence of new features in the vibrations of the partially crystalline phase of Pb_3_TeO_6_. The existence of Pb_3_TeO_6_ is confirmed by X-ray analysis. Clearly, the shoulders at 410 cm^−1^ were getting lower as the irradiation dose increased, possibly due to the network compaction. The spectral features from 710 cm^−1^ to 730 cm^−1^ and 790 cm^−1^ correspond to the TeO_4_ bi-pyramidal arrangement and the TeO_3+1_ (or distorted TeO_4_) and TeO_3_ trigonal pyramids structures respectively. It can be clearly observed that the evolution of TeO_3+1_ and TeO_3_ units occurs as the TeO_2_ concentration decreases. Legitimately, the decreasing peak on the TeO_4_ bi-pyramidal arrangement and TeO_3+1_ (or distorted TeO_4_) is due to augmentation of irradiation dose which is attributed to an increase in degree of disorder of the amorphous phase. According to El-Alaily and Mohamed [3], irradiation with gamma rays are assumed to create displacements, electronic defects and/or breaks in the network bonds, which allow the structure to relax and fill the relatively large interstices that exist in the interconnected network of boron and oxygen atoms causing expansion followed by compaction of the volume. Shelby [13] also suggested that the boron-oxygen bond is more likely to be affected by irradiation.

### 2.2. XRD

**Figure 3:**
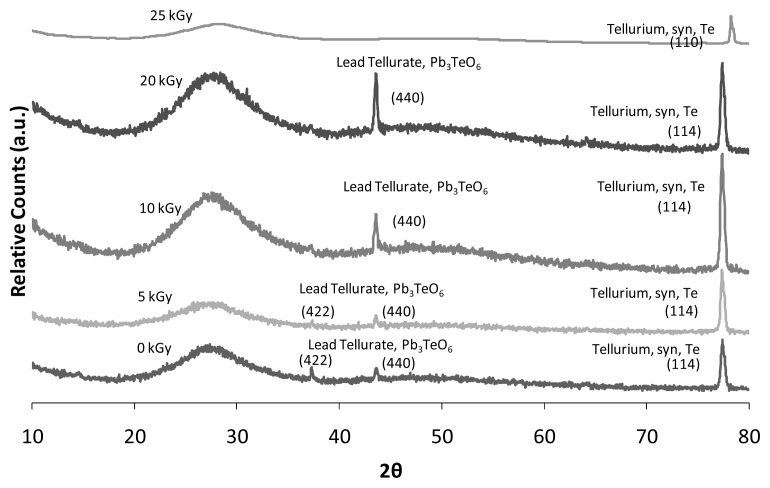
X-ray diffractogram patterns (at ambient temperature) of (TeO_2_)*_y_*[(PbO)*_x_*(B_2_O_3_)_1−_*_x_*]_1-_*_y_* glasses (*x* = 0.5% mol; *y* = 0.7% mol) with varied gamma irradiation dose.

The XRD diffractograms result in Figure 3 shows the partially crystalline precipitation before and after irradiation exposure for *x* = 0.5% mol; *y* = 0.7% mol. All the glass that was prepared proved to fit the amorphous state. In addition to the enhanced amount of PbO, the glass had been acclimatized to the partially crystalline phase from the full amorphous phase. It also shows the presence of a hunch for 2θ around 20°–35°. All these XRD reflections were assigned to the two polymorphic phases; hexagonal of tellurium (Te, PDF2 No. 00-036-1402) and monoclinic of lead tellurate (Pb_3_TeO_6_, PDF2 No. 00-033-0770). The intensity of the XRD reflections indicate that more monoclinic crystals are reduced in quenched samples as the modifier content increases. The structures of lead tellurate contain Pb_3_TeO_6_ probably consisting of TeO_3_ trigonal pyramid connected by PbO_4_ tetragonal, forming a three-dimensional network. The decrease of glass rigidity is due to irradiation processes which are supported by the XRD diffractogram results in Figure 3. The XRD result shows the presence of crystalline precipitation before irradiation process. However, the partially crystalline peaks vanished due to 5 kGy of gamma irradiation exposure. Eventually, modification of lattice and structural arrangement along with network compaction occurs in the system due to irradiation exposure of more than 5 kGy which comprises the presence of crystalline peaks. As the irradiation dose increases (above 20 kGy), the crystalline peaks once again begin to diminish. Obviously, the partially crystalline glass still proved to fit the amorphous state as the irradiation dose increased.

### 2.3. Optical Absorption Spectra

**Figure 4:**
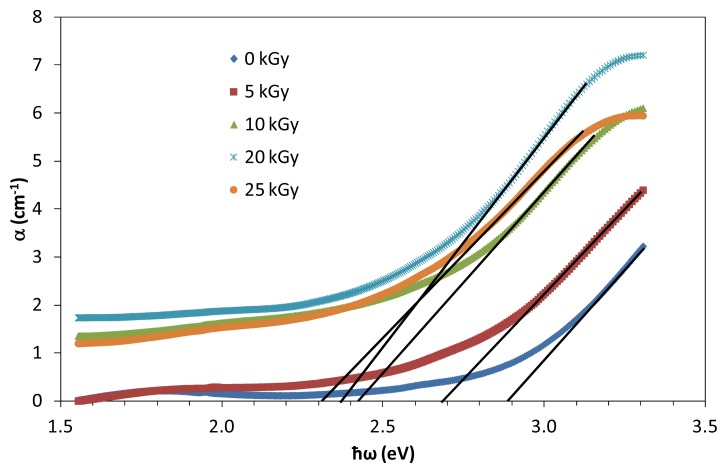
Optical absorption edge, α of (TeO_2_)*_y_*[(PbO)*_x_*(B_2_O_3_)_1−_*_x_*]_1−_*_y_* glasses with *y* = *x* = 0.5% mol; 0.7% mol at various irradiation exposure. The lines represent the expolation.

The optical absorption spectra were taken in the ranges of 340 to 550 nm. The optical absorption is one of the most productive tools to understand the band gap of optical materials. The optical properties of a solid are governed by the interaction between the solid and the electric field of the electromagnetic wave. The optical absorption measurements coefficient α(ω) near the fundamental is calculated from absorbance *A*, using the following Equation [14]:

(2) α(ω)=2.303 A/d

where *d* is the thickness of the samples. The rapid change in α(ω) against ω is called “the fundamental absorption edge” and the corresponding energy is defined as “the optical energy gap (*E_opt_*). In the compound, a typical absorption edge can be broadly ascribed to any of the three processes: (i) residual below-gap absorption; (ii) Urbach tails; and (iii) interband absorption. In the second process, the absorption edge depends exponentially on the photon energy according to the Urbach relation. In crystalline materials the fundamental edge is directly related to the conduction band and valence band, *i.e.*, direct and indirect band gaps, while in the case of amorphous materials a different type of optical absorption edge is observed. Figure 4 illustrates the variation of absorption coefficient, α with incident photon energy at different doses.

**Figure 5:**
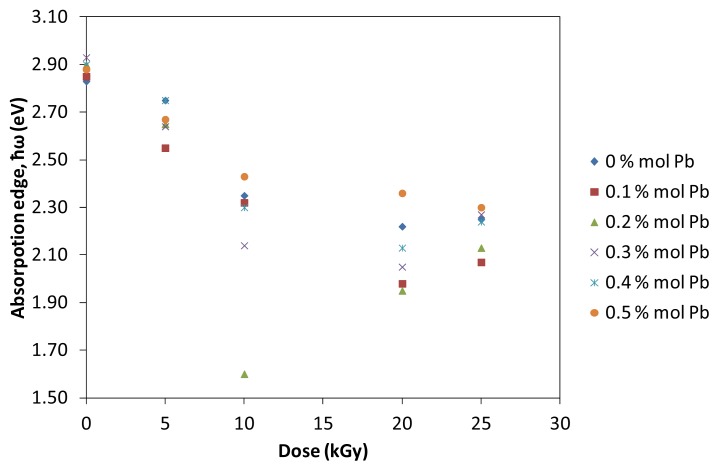
The dependence of Urbach’s absorption edge on different irradiation dose for (TeO_2_)*_y_*[(PbO)*_x_*(B_2_O_3_)_1−_*_x_*]_1−_*_y_* glasses with *y* = *x* = 0.5% mol; 0.7% mol.

Urbach edge analysis is a useful way to parametrically characterize glass optical absorption edge and potentially distinguished intrinsic contributions to absorbance. Urbach’s absorption edge is formed in the region of photon energies below the forbidden gap. The interaction between lattice vibrations and localized states in tail of band gap from the glass samples has a significant effect on the optical properties. The plot of ln (α) against photon energy, ħω is linear for the absorption region near the fundamental absorption edge. Thus, it is evaluated that the absorption coefficient near the fundamental absorption edge is exponentially dependent on the photon energy and obeys the Ubach’s rule. Figure 5 illustrates the dependence of Urbach’s absorption edge with different irradiation of prepared glasses. The absorption edge decreases with the increase of dose. Significantly, the decreasing values of absorption edge indicate that after irradiation processes, there is red shift effect on the (TeO_2_)*_y_*[(PbO)*_x_*(B_2_O_3_)_1−_*_x_*]_1−_*_y_* glasses. The red shift effect is a process when the absorbance band shifts to longer wavelength and widens due to irradiation. A shift in the absorption edge can be attributed to an increase of the conjugation length. The number of Te atoms and Pb atoms per conjugation length is found to increase with increasing dose which create structure defect within the prepared (TeO_2_)*_y_*[(PbO)*_x_*(B_2_O_3_)_1−_*_x_*]_1−_*_y_* glass.

**Figure 6:**
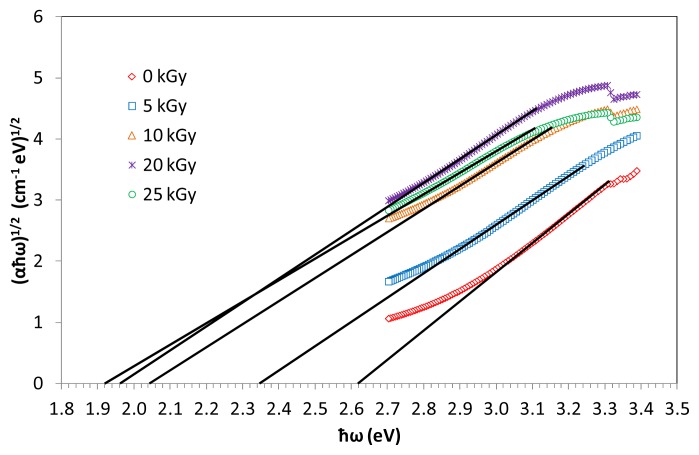
Plot of (αħω)^1/2^ against photon energy for indirect band gap of (TeO_2_)*_y_*[(PbO)*_x_*(B_2_O_3_)_1−_*_x_*]_1−_*_y_* glasses with *y* = *x* = 0.5% mol; 0.7% mol at various irradiation exposure. The lines represent the pattern.

The optical band gap energy is determined by using the following Equation [15]

(3) $$\alpha \hbar \omega =A{\left(\hbar \omega -E_{opt}\right)}^{n}$$

where α is the absorption coefficient, ħω is the incident photon energy, *A* is a constant and *E*_opt_ is the optical band gap. Values of *n* are 2 and 1/2 for direct and indirect transitions, respectively. Figure 6 shows the information of indirect band gap (αħω)^1/2^ against photon energy ħω of (TeO_2_)*_y_*[(PbO)*_x_*(B_2_O_3_)_1−_*_x_*]_1−_*_y_* glasses with *x* = 0.5 mol %; *y* = 0.7 mol % at various irradiation exposure, plotted in the absorption region. Indirect energy gap is determined from the linear regions of the plots as shown in the figures and corresponding values presented in Table 2. The *E_op_*_t_ has been calculated approximately from the linear region of the arc extrapolating to meet the ħω axis at (αħω)^1/2^*=*0.

**Figure 7:**
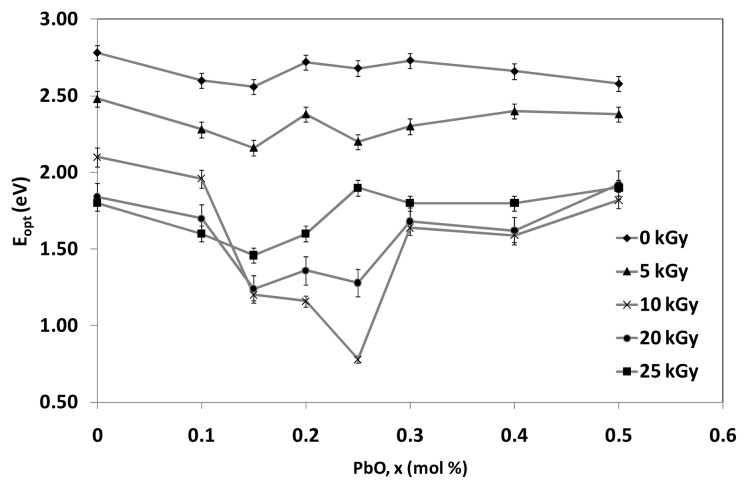
Variation optical band gap, *E*_opt_ of (TeO_2_)*_y_*[(PbO)*_x_*(B_2_O_3_)_1−_*_x_*]_1−_*_y_* glasses for indirect transition with *x* = 0%–0.5% mol; *y* = 0.7% mol at various irradiation exposure. The lines represent the extrapolation.

The variation of indirect optical band gap with mole fraction of PbO content before and after irradiation is shown in Figure 7. The connected lines do not resemble any significant explanations rather than to show the decreasing and increasing pattern of the graph. The optical band gap values of the indirect process before irradiation decreases through the augment of PbO from 0% mol to 0.15% mol. This is due to the increase of the network disorder and consequently the extension of the localized states within the gap. More likely, PbO enters the glass network by breaking up the Te–O–Te and B–O–B bonds and introduces coordinate defects known as dangling bonds along with non-bridging oxygen. Normally, the oxygen of PbO breaks the local symmetry while Pb^2+^ ions occupy interstitial positions. PbO content >0.2% mol shows a slight increase before tends to decline slowly for 0 kGy and increase for irradiated samples with 5 kGy up to 25 kGy (towards 0.5% mol). Consequently, as PbO increases (from 0.2% to 0.5% mol), modification of lattice and interstitial occurs in the system with the nearest neighboring atoms and arrangements, such as PbO_4_ and/or BO_4_. A considerable portion may act as double bridges between adjacent TeO_4_ which can form in addition to the formation of PbO_4_ and TeO_3_ units. Therefore, for PbO ≥ 0.2% mol, Pb^2+^ acts as glass forming agent and is incorporated in the glass network in the form of PbO_4_ units. The optical band gap, *E*_opt_ values for indirect transition decrease with increasing of irradiation dosage as the content of PbO ≤ 0.2% mol due to increase in degree of disorder of the amorphous phase. Obviously, the *E*_opt_ values for indirect transition increase with increasing of irradiation dosage as the content of PbO > 0.2% mol. It is believed that the increasing of irradiation create displacements, electronic defects and/or breaks in the network bonds, which allow the structure to relax and fill the relative large interstices that exist in the interconnected network of boron and oxygen atoms causing expansion followed by compaction of the volume.

**Figure 8:**
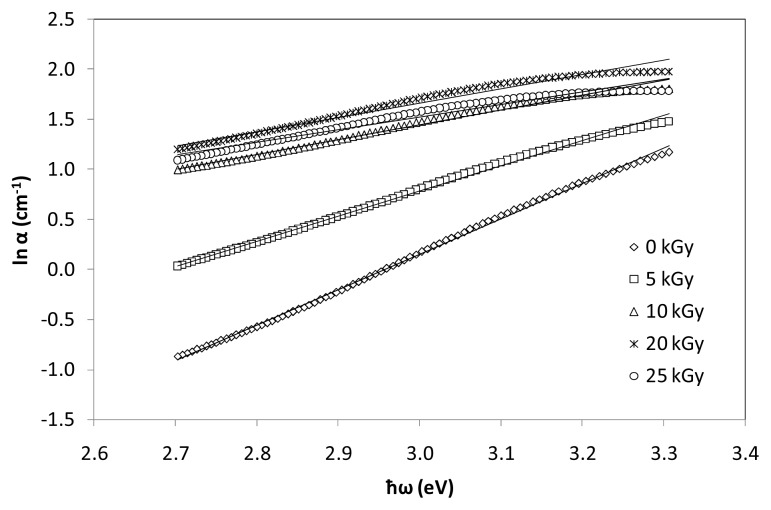
Optical absorption coefficient of (TeO_2_)*_y_*[(PbO)*_x_*(B_2_O_3_)_1−_*_x_*]_1−_*_y_* glasses with *y* = *x* = 0.5% mol; 0.7% mol at various irradiation exposure. The line represents the pattern.

**Figure 9:**
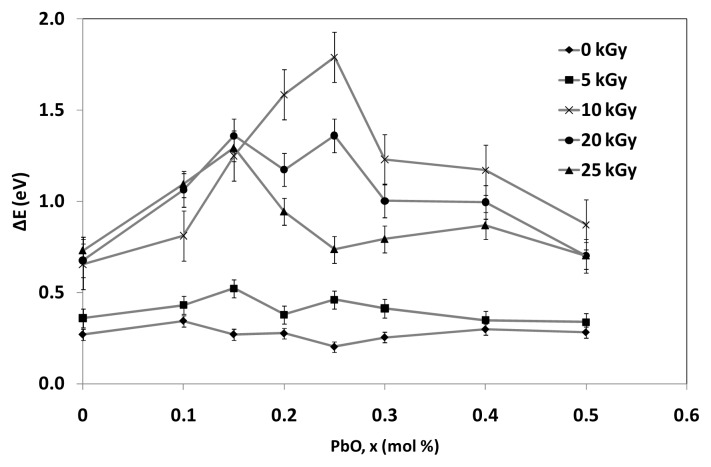
Variation Urbach energy, Δ*E* of (TeO_2_)*_y_*[(PbO)*_x_*(B_2_O_3_)_1−_*_x_*]_1−_*_y_* glasses for indirect transition with *x* = 0%–0.5% mol; *y* = 0.7% mol at various irradiation exposure. The lines represent the pattern.

In many crystalline and non-crystalline semiconductors, the α(ω) depends exponentially on the ħω. This exponential dependence, known as the Urbach rule, can be written in the form [16]:

(4) $$\alpha (\omega )=Bexp\left(\frac{\hbar \omega }{\Delta E}\right)$$

where B is a constant and Δ*E* is the width of the band tails of the localized states which also known as Urbach energy. The value of Urbach energy (Δ*E*) is calculated by taking the reciprocals of the slopes of the linear portion of the ln α(ω) against ħω curves in the lower photon energy regions. Figure 8 shows the reciprocal of the slopes of the linear portion from the ln α(ω) against ħω curves in the lower photon energy regions for (TeO_2_)*_y_*[(PbO)*_x_*(B_2_O_3_)_1−_*_x_*]_1−_*_y_* glasses with *x* = 0.5% mol; *y* = 0.7% mol at various irradiation exposure. The reciprocal values will be used to calculate the value of Urbach energy (Δ*E*) using the Equation 3. The values of (Δ*E*) of (TeO_2_)*_y_*[(PbO)*_x_*(B_2_O_3_)_1−_*_x_*]_1−_*_y_* glasses’ various irradiation exposure with *x* = 0.0%–0.5% mol; *y* = 0.7% mol are visualized in Figure 9 and tabulated in Table 2. Generally, an increase and decrease in Urbach energy can be considered as being due to defects within the glass network.

Urbach’s energy is a characteristic energy which determines how rapidly the absorption coefficient decreases for below band gap energy. Urbach measured the absorption tail for different temperatures and showed that the Urbach’s energy is approximately *kT*, the thermal energy. The temperature dependence of the urbach tail led to the conclusion that the below-bandgap transitions are assisted transition. The Urbach tail can be caused by mechanisms other than phonon-assisted absorption. Considering Figure 9, before irradiation took place, the Ubach energy having a non-prominent increase pattern when the content PbO in the network increase which illustrating the saturation of defects at high content of PbO. Other than that, it is more likely due to the increase of the disorder and consequently the more extension of the localized states. For every irradiation dose of between 5 kGy and 25 kGy, Ubach energy increases as the content of PbO ≤ 0.2% mol suggesting the increasing of disorder and consequently the further extension of the localized states. Nevertheless, Ubach energy for every irradiation dose from 5 kGy up to 25 kGy decreases as the content of PbO > 0.2% mol advocating the possibility of long range order locally arising from the minimum in the number of defects.

With the increasing of irradiation dosage, the content of PbO ≤ 0.2% mol, the Ubach energy increases significantly, this suggests the increase in degree of disorder of the amorphous phase. As the content of PbO increases to be greater than 0.2% mol, the Ubach energy increases with 5 kGy and 10 kGy of irradiation dose and begins to decrease with irradiation dosage of 20 kGy and 25 kGy. It is believed that the increasing of irradiation creates displacements, electronic defects and/or breaks in the network bonds, which allow the structure to relax and fill the relative large interstices that exist in the interconnected network, causing expansion followed by compaction of the volume.

## 3. Experimental Section

The ternary (TeO_2_)*_y_*[(PbO)*_x_*(B_2_O_3_)_1−_*_x_*]_1−_*_y_* glass system (*x* = 0.0%–0.50% mol and *y* = 0.7% mol) were prepared using a conventional melt-quenching method [17]. All the glass samples arranged were homogenous, transparent and bubble free. The glasses were prepared by mixing together specific weights of Tellurium dioxide—TeO_2_ (Alfa Aesar 99.99%), Lead oxide, Litharge—PbO (99%) and Boron oxide—B_2_O_3_ (Alfa Aesar, 97.5%). Appropriate amounts of TeO_2_, PbO and B_2_O_3_ were weighed by using an electronic balance having an accuracy of 0.0001 g. The chemicals were then thoroughly mixed in an Agate pestle mortar for half an hour and poured into an Alumina crucible. The crucible was transferred to a furnace and heated at 950 °C for 2 h to aid the melting process. When the melting process was complete, the molten liquid was cast into a stainless steel cylindrical shape mold which had been preheated at 340 °C for 30 min. The produced glass samples were annealed at the temperature range 340 °C for 2 h, and then the furnace was turned off for cooling process reaching the atmospheric temperature. The glass samples were cut using Buhler ISOMET diamond cutter at a thickness of approximately 2 mm for the required measurements.

The irradiation process had been conducted using ^6^°Co gamma rays (J.L. Sherperd & Associates, model 109-68# 3044) with the dose rate of 5.52 kGyh^−1^ on March 2004. The ^6^°Co radioisotope produced two gamma rays of energy 1.17 MeV and 1.33 MeV, often indicated in the machine as the average energy of 1.25 MeV, is the most widely used gamma source not only for research, but also for the food preservation, sterilization of medical equipment and pharmaceutical raw materials. The dose rate at the day of irradiation was calculated using decay equation *D_t_* = *D*_o_*e*^(−λt)^ where λ is the decay constant, *D_t_* is the dose rate at time *t* and *D_0_* is the dose rate calibrated using Fricke dosimeter (Ferrous Sulphate). The half-life of ^6^°Co is 5.3 years. The irradiation has been conducted at the building 41, SINAGAMA at Malaysia Nuclear Agency. Raman spectra were measured using a Raman spectrometer (RSI 2001 B, Raman system, INC) equipped with a 532 nm solid-state diode green laser. Grams/32, version 6 software was used to analyze the spectra. All spectra were corrected for baseline; smoothed and Fourier Transformed (FT). The baseline correction utilized the multiple point level method in which the baseline is leveled at a value that is the average of the baseline points. A constant correction factor of 80% of the degree of smoothing parameter was used throughout the data collection. The Fourier smoothing was accomplished by the peak data, applying a triangular filter function at the specified cut-off point of 40% and then reverse Fourier transforming the data. Optical absorption measurements in the wavelength range of 190 to 800 nm with 0.1 nm internal spacing at slow scan speed were performed at room temperature using SHIMADZU spectrophotometer model UV-1650PC (absorption within ± 0.1 a.u. and wavelength within ± 2 cm^−1^). Phase identification of the samples will be determined by X-ray Powder Diffractometer, Philip X’Pert Pro Holland, using Cu-K_α_ monochromatized radiation (λ = 1.5418 mm). The operating generator tension is 40 kV while generator current is 30 mA. X-Ray diffraction patterns of powders were recorded at room temperature with a diffraction angle from 2θ = 5°–90° and at a rate of 0.01°/min. The XRD measurements were held using bulk dimension since the results gained did not shows any significant difference between bulk dimension and powder dimension.

## 4. Conclusions

The structural and optical properties of lead borotellurite glass system were studied as model glasses for radiation protection material. They depend not only on the content of Tellurium dioxide as the network former, but rather on the total fraction of network modifying oxides (PbO, B_2_O_3_) in the glass structure and effect of radiation towards the glass network. The increasing peak on the TeO_4_ bi-pyramidal arrangement and TeO_3+1_ (or distorted TeO_4_) is due to augmentation of irradiation dose which is attributed to an increase in degree of disorder of the amorphous phase. The existence of Pb_3_TeO_6_ in Raman spectra is confirmed by X-ray analysis. XRD shows the presence of a hunch for 2θ around 20°–35°. The structures of lead tellurate contain Pb_3_TeO_6_ probably consisting of TeO_3_ trigonal pyramid connected by PbO_4_ tetragonal forming a three-dimensional network. The decrease of glass rigidity is due to irradiation processes which supported by the XRD diffractograms results. Significantly, the decreasing values of absorption edge indicate that red shift effect occur after the irradiation processes. Red shift effect is a process when the absorbance band shifts to longer wavelength and widens due to irradiation. A shift in the absorption edge can be attributed to an increase of the conjugation length. The values of optical band gap, *E*_opt_ were calculated and found to be dependent on the glass composition and radiation exposure. The increasing of irradiation creates displacements, electronic defects and/or breaks in the network bonds, which allow the structure to relax and fill the relative large interstices that exist in the interconnected network causing expansion followed by compaction of the volume.

## Figures and Tables

**Table 1:** Marker labeling Raman peak according to chemical bonding and stretching [12].

Bonding	Raman Shift (cm^−1^)	
S—Strong	(S) <100cm^−1^;	Pb, Te
W—Weak	(S) 450 cm^−1^;	B–O–B (b)
B—Bending	410 cm^−1^;	
G—Group	(W) 490 cm^−1^;	Te–O–Te (ss)
as—Asymmetry stretching	(S) 710 cm^−1^ ;	TeO^4^ bi-pyramidal arrangement
ss—Symmetry stretching	(S) 730 cm^−1^;	TeO^3+1^ (distorted TeO^4^)
	(S) 790 cm^−1^–860 cm^−1^;	Te–O bending vibrations in TeO^3^ trigonal pyramids and TeO^6^

**Table 2:** Optical characteristic for (TeO_2_)*_y_*[(PbO)*_x_*(B_2_O_3_)_1−_*_x_*]_1−_*_y_* glasses, *y* = 0.7% mol.

PbO, *x* (mol %)	Dose (kGy)	Direct transition *E*_opt_ (eV)	Indirect transition *E*_opt_ (eV)	Urbach energy Δ*E* (eV)
0.00	0	3.22	2.78	0.27
	5	3.13	2.48	0.36
	10	2.82	2.10	0.65
	20	2.76	1.84	0.68
	25	2.74	1.80	0.73

0.10	0	3.22	2.78	0.27
	5	2.97	2.28	0.43
	10	2.82	1.96	0.81
	20	2.72	1.70	1.06
	25	2.62	1.60	1.09

0.20	0	3.18	2.72	0.28
	5	2.98	2.38	0.38
	10	2.54	1.16	1.58
	20	2.62	1.36	1.17
	25	2.66	1.60	0.94

0.30	0	3.16	2.73	0.25
	5	2.94	2.30	0.41
	10	2.66	1.64	1.23
	20	2.68	1.68	1.00
	25	2.76	1.80	0.79

0.40	0	3.16	2.66	0.30
	5	3.04	2.40	0.35
	10	2.74	1.59	1.17
	20	2.78	1.62	1.00
	25	2.80	1.80	0.87

0.50	0	3.10	2.58	0.28
	5	3.00	2.38	0.34
	10	2.80	1.82	0.87
	20	2.82	1.92	0.70
	25	2.92	1.90	0.70
